# Amylin and pramlintide modulate γ-secretase level and APP processing in lipid rafts

**DOI:** 10.1038/s41598-020-60664-5

**Published:** 2020-02-28

**Authors:** Youssef M. Mousa, Ihab M. Abdallah, Misako Hwang, Douglas R. Martin, Amal Kaddoumi

**Affiliations:** 1 Department of Drug Discovery and Development, Harrison School of Pharmacy, Auburn University, Auburn, USA; 2 Scott-Ritchey Research Center, Auburn University, Auburn, USA; 3 Department of Anatomy, Physiology and Pharmacology, College of Veterinary Medicine, Auburn University, Auburn, USA; 4 Center for Neuroscience Initiative, Auburn University, Auburn, AL USA

**Keywords:** Molecular neuroscience, Molecular neuroscience, Translational research, Translational research

## Abstract

A major characteristic of Alzheimer’s disease (AD) is the accumulation of misfolded amyloid-β (Aβ) peptide. Several studies linked AD with type 2 diabetes due to similarities between Aβ and human amylin. This study investigates the effect of amylin and pramlintide on Aβ pathogenesis and the predisposing molecular mechanism(s) behind the observed effects in TgSwDI mouse, a cerebral amyloid angiopathy (CAA) and AD model. Our findings showed that thirty days of intraperitoneal injection with amylin or pramlintide increased Aβ burden in mice brains. Mechanistic studies revealed both peptides altered the amyloidogenic pathway and increased Aβ production by modulating amyloid precursor protein (APP) and γ-secretase levels in lipid rafts. In addition, both peptides increased levels of B4GALNT1 enzyme and GM1 ganglioside, and only pramlintide increased the level of GM2 ganglioside. Increased levels of GM1 and GM2 gangliosides play an important role in regulating amyloidogenic pathway proteins in lipid rafts. Increased brain Aβ burden by amylin and pramlintide was associated with synaptic loss, apoptosis, and microglia activation. In conclusion, our findings showed amylin or pramlintide increase Aβ levels and related pathology in TgSwDI mice brains, and suggest that increased amylin levels or the therapeutic use of pramlintide could increase the risk of AD.

## Introduction

Alzheimer’s disease (AD) is a complex neurodegenerative disorder characterized by multiple dysregulated neurobiological networks and cellular functions, neuronal death, and memory loss^1^. The major hallmarks for AD include amyloid-β (Aβ) deposits and neurofibrillary tangles of hyper-phosphorylated tau protein^1,2^. Aβ is produced from the amyloid-β precursor protein (APP), which, after synthesis, traffics to the Golgi apparatus and eventually to plasma membrane^3^. The majority of plasma membrane bound APP is rapidly endocytosed^3^, while approximately 10% of the total APP is processed in the plasma membrane by α-secretase to release soluble APP-α (sAPP-α)^4^. Alternatively, APP can be processed by β-site APP cleaving enzyme 1 (BACE1) in plasma membrane, trans-Golgi, and early endosomes to produce soluble APP-β (sAPP-β) and β-C-terminal fragments (β-CTF), followed by subsequent proteolytic cleavage of β-CTF by γ-secretase to release Aβ^4^. Besides Aβ production, another contributing factor to Aβ accumulation in AD is the failure to clear Aβ from the brain^5^, and across the blood-brain barrier (BBB)^6^.

The accumulation of Aβ due to decreased clearance or increased production leads to multiple dysregulated cellular functions including synaptic loss and neuroinflammation^7,8^. Aβ initiates synaptic loss by down-regulating synaptic markers in AD including the pre-synaptic marker SNAP-25 and synapsin I and post-synaptic marker PSD-95^9^. Moreover, the accumulation of Aβ in the brain parenchyma of AD patients is associated with strong inflammatory processes and glial cells activation^7^.

Mounting evidence supports the localization of APP, BACE1 and each of the four core subunits of the γ-secretase complex in detergent resistant membranes (DRMs), microdomain fractions that are positive for lipid rafts markers^10–12^. These rafts are rich in sphingolipids and cholesterol^13^. Lipid rafts are small, highly dynamic and heterogenous domains that compartmentalize cellular processes such as pathogen entery, motility, cell adhesion, cell signaling, trafficking and protein sorting^13^. The rafts are rich in GM1 ganglioside which is the most abundant ganglioside in the brain^14^. The interactions between APP and the membrane associated secretases are believed to occur at multiple cellular compartments containing GM1^15^. Much research activity over the past two decades has shown that GM1 is involved in the pathogenesis of AD by increasing the production of Aβ through modulating the activity of γ-secretase and APP localization in lipid rafts^16,17^.

Type 2 diabetes (T2D) has been identified as a risk factor for AD^18^, and both diseases are pathologically characterized by the presence of misfolded protein aggregates, which result in the formation of Aβ deposits in AD and amylin deposits in T2D^19^. Amylin (also known as islet amyloid polypeptide) is a 37- amino acid peptide that is co-secreted with insulin from the pancreatic-β cells in response to food intake and works as a satiating hormone^20^. The misfolded amylin was first isolated from amyloid-rich pancreatic extracts from T2D patients^21^ in the form of elongated fibrils with many stranded β sheets^22^. In addition to the formation of amylin deposits in the pancreas, these deposits are found in the brains of AD patients with T2D^23^. Amylin crosses the BBB and it is suggested to have a role in neural regeneration and glucose metabolism^24^. Amylin and Aβ have several common features such as having similar β-sheet structures^25^, binding to the same amylin receptor^26^, and being degraded by insulin degrading enzyme (IDE)^27^.

Several studies have shown that amylin is involved in the pathogenesis of AD by inducing neuroinflammation and apoptosis^28^, but little is known about the mechanism by which amylin exacerbates AD pathology. On the other hand, multiple studies have shown that amylin ameliorated AD pathology by decreasing neuroinflammation and increasing Aβ clearance from brain to blood^29–32^. In this study, we investigated the effect of amylin and its alanog, pramlintide, on Aβ pathology, and identified a novel aspect of amylin and pramlintide in increasing the amyloidogenic processing of APP in TgSwDI mouse, a model for cerebral amyloid angiopathy (CAA) and AD, where both peptides increased γ-secretase complex level and APP localization in lipid rafts. Furthermore, pramlintide significantly increased GM1 ganglioside in lipid rafts and GM2 in total brain homogenate, whereas both peptides increased the ganglioside producing enzyme β-1,4 N-acetylgalactosaminyl transferase 1 (B4GALNT1). Our findings suggest that amylin and pramlintide increased Aβ brain accumulation through modulating APP processing in lipid rafts, and thus could increase the risk of CAA and AD.

## Results

### Amylin and pramlintide did not alter memory performance compared to vehicle treated mice

Morris water maze (MWM) behavioral test was performed to assess the effect of amylin and pramlintide on learning and memory functions. The following parameters were analyzed, the time a mouse takes to find the platform (latency, s), swimming speed (cm/s), swimming distance (cm) and number of entries in target quadrant. As shown in Fig. S1 (Supplementary Material), amylin and pramlintide have no significant changes in the evaluated parameters when compared to vehicle treated mice (control), suggesting amylin and pramlintide did not alter memory function.

### Amylin or pramlintide increased Aβ burden in TgSwDI mice brains

**Figure 1:**
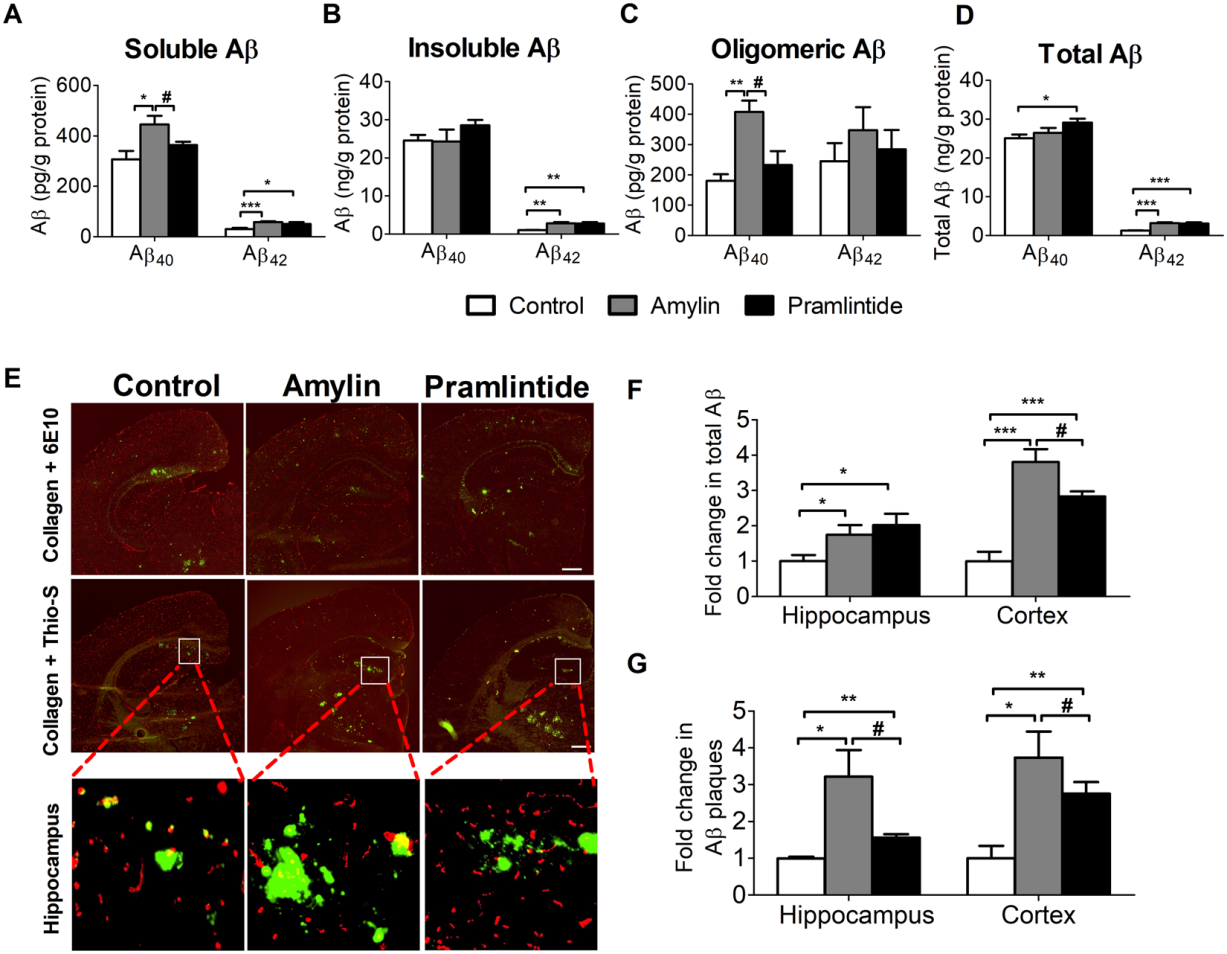
Effect of amylin and pramlintide treatments on Aβ burden in TgSwDI mice brains. **(A)** Using ELISA to quantify the different forms of Aβ, amylin increased the level of soluble Aβ_40_ and Aβ_42_ compared to control and pramlintide, whereas pramlintide increased the level of soluble Aβ_42_ compared to control group. **(B)** Both peptides showed significant increase of insoluble Aβ_42_ compared to control when measured by ELISA; however, none of the treatments changed the level of insoluble Aβ_40_ (**C**) Amylin significantly increased oligomeric Aβ_40_ compared to control and pramlintide, but neither treatment altered the oligomeric Aβ_42_. **(D)** Both peptides showed significant increase of total Aβ_42_ compared to control when measured by ELISA; while pramlintide only increased levels of total Aβ_42_. **(A**–**C)** Aβ level was normalized to the total protein content in the measured samples as measured by ELISA. **(E**–**G)** The IHC analysis demonstrated a significant increase in total Aβ (detected by 6E10, green color) in the brains of both amylin and pramlintide compared to control when measured in the cortex and hippocampus. Moreover, amylin significantly increased the level of total Aβ in the hippocampus compared to the pramlintide group. Quantification analysis demonstrated Aβ plaques (detected by Thioflavin-S, green color) were significantly higher in hippocampus and cortex of amylin and pramlintide treated mice compared to control group. Brain microvessels are stained by collagen IV antibody (red). Scale bar = 500 µm. Data is presented as mean ± SEM for n = 4 per group for figures (**A–D**) and n = 3 per group for figures. (**E–G**) The statistical significance for all result was assessed by student t-test, with *p < 0.05, **p < 0.01, and ***p < 0.001 compared to control group, and ^#^*p* < 0.05 compared to pramlintide.

Aβ levels from brain total homogenate were analyzed by ELISA and the results demonstrated that only amylin increased the level of soluble Aβ_40_ compared to control (*p* < 0.05), while both amylin and pramlintide significantly increased soluble Aβ_42_ compared to control group (*p* < 0.001 and *p* < 0.05, respectively) (Fig. 1A). Moreover, neither amylin nor pramlintide treatment showed significant changes in the level of insoluble Aβ_40_, but both showed significant increase in insoluble Aβ_42_ compared to control (*p* < 0.01; Fig. 1B). Furthermore, amylin significantly increased oligomeric Aβ_40_ compared to control and pramlintide (*p* < 0.01 and *p* < 0.05, respectively); however, neither treatment altered oligomeric Aβ_42_ (Fig. 1C). Total Aβ_40_ and Aβ_42_ from total protein fraction were also measured following direct extraction with 70% formic acid; as shown in Fig. 1D, amylin significantly increased total levels of Aβ_42_ only (*p* < 0.001), while pramlintide increased both Aβ_40_ (*p* < 0.05) and Aβ_42_ (*p* < 0.001) when compared to control group.

Immunohistochemical analysis of the three groups was performed to show Aβ burden in cortex and hippocampus regions. The captured images showed a significant increase in total Aβ (detected by 6E10) in the brains of both amylin and pramlintide treated mice compared to control when measured in the hippocampus (*p* < 0.05), and cortex (*p* < 0.001; Fig. 1E–G). Moreover, the deposition of Aβ plaques (detected by Thio-S) was significantly higher in the brains of mice treated by amylin or pramlintide than the control group in the hippocampus (*p* < 0.05 and *p* < 0.01, respectively) and cortex (*p* < 0.05 and *p* < 0.01, respectively) (Fig. 1E–G).

### Amylin and pramlintide have no clear effect on APP processing pathway when measured in brain homogenate

**Figure 2:**
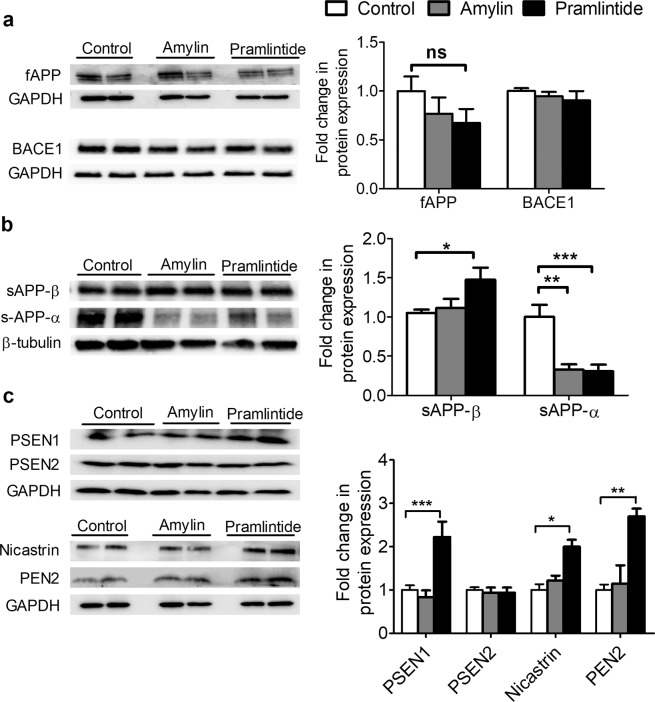
Effect of amylin and pramlintide on APP processing in total brain homogenate. **(a)** Representative Western blot and densitometry analysis of full-length APP (fAPP) and BACE1 demonstrated amylin and pramlintide did not alter full-length APP (fAPP) and BACE1 in mice brain homogenates. The fAPP and BACE1 levels were normalized to GAPDH level. **(b)** Representative Western blot and densitometry analysis of sAPP-β and sAPP-α in mice brains demonstrated pramlintide significantly increased sAPP-β compared to control, whereas reduction in the level of sAPP-α was observed after treatment with amylin and pramlintide. The levels of sAPP-β and sAPP-α were normalized to the level of β-tubulin. sAPP-β and sAPP-α were ran on different gels due to molecular weight similarity. **(c)** Representative Western blot and densitometry analysis of γ-secretase subunits in mice brains demonstrated pramlintide caused a significant increase in PEN2 subunit when compared to control and amylin; however, neither peptide influenced the other γ-secretase subunits PSEN1, PSEN2 and nicastrin. The level of γ-secretase subunits was normalized to level of GAPDH in each corresponding lane. PSEN1 and PSEN2 were ran on different gels due to molecular weight similarity. **(d)** Representative Western blot and densitometry analysis of β-CTF in mice brains demonstrated amylin and pramlintide caused a significant increase in β-CTF when compared to control. Data is presented as mean ± SEM, and the densitometry analysis is from n = 6 mice in each group. The western blot results are representative results from two different mice from each group. Data is presented as mean ± SEM and the statistical significance for all result was assessed by student t-test, with *p < 0.05, **p < 0.01, ***p < 0.001 compared to control group; ^#^*p* < 0.05 compared to pramlintide, and ns = not significant.

Findings from Western blot of mice brain homogenates demonstrated insignificant changes in the level of full-length APP (fAPP) and BACE1 between control and treated mice (Fig. 2a). The cleavage of APP by BACE1 produces sAPP-β and the results demonstrated that only pramlintide significantly increased sAPP-β compared to control (*p* < 0.05), whereas both amylin and pramlintide significantly decreased the level of sAPP-α in the total brain homogenate compared to control (both, *p* < 0.01; Fig. 2b). To further understand the effect of amylin and pramlintide on APP processing and to explain the increased Aβ burden in brain homogenates, the four γ-secretase complex subunits, presenilin-1 (PSEN1), presenilin-2 (PSEN2), nicastrin, and PEN2 were measured. Results from the total brain homogenate showed no significant changes in PSEN1, PSEN2, and nicastrin between the control and treated mice (Fig. 2c). On the other hand, only pramlintide demonstrated a significant increase in PEN2 subunit when compared to control and amylin (*p* < 0.001 and *p* < 0.05, respectively) (Fig. 2c). Next, to explain whether increased brain Aβ is mediated by γ-secretase, we evaluated levels of β-CTF (or C99) in brain homogenates by Western blotting, and our findings showed both amylin and pramlintide significantly increased β-CTF levels compared to control (*p* < 0.001 and *p* < 0.05, respectively) (Fig. 2d). Overall, the results from the total brain homogenate did not provide clear explanation for the increased Aβ burden in the brains of mice treated with amylin and pramlintide.

### Amylin and pramlintide modulated APP processing in lipid rafts

**Figure 3:**
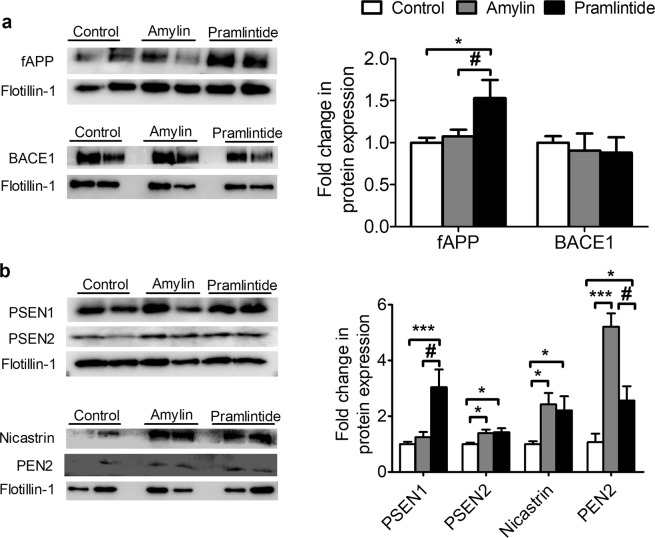
Effect of amylin and pramlintide on APP processing in lipid rafts. **(a)** Representative Western blot and densitometry analysis of fAPP and BACE1 in lipid rafts demonstrated pramlintide significantly increased APP in lipid rafts compared to control. The level of BACE1 in lipid rafts did not change between the three groups. **(b)** Representative Western blot and densitometry analysis of γ-secretase subunits in lipid rafts demonstrated amylin and pramlintide showed significant increase in the level of PSEN1, PSEN2, nicastrin and PEN2 compared to the control group. In addition, amylin increased the level of PEN2 compared to pramlintide when measured from lipid rafts. PSEN1 and PSEN2 were ran on different gels due to molecular weight similarity. In the figures (**a,b**), the measured proteins were normalized to the level of flotillin-1 in each corresponding lane, and the densitometry analysis is from n = 6 mice in each group. The western blot results are representative results from two different mice from each group. Data is presented as mean ± SEM and the statistical significance for all result was assessed by student t-test, *p < 0.05 compared to control group; ^#^*p* < 0.05 com*p*ared to pramlintide.

Mounting evidence indicates that the amyloidogenic processing of APP occurs in lipid rafts^10–12^. The DRMs were prepared and fractions enriched in lipid rafts were identified by immunoblotting of lipid raft marker with antibody against flotillin-1. Findings from optimization and characterization of lipid rafts isolation from brain homogenates demonstrated the highest flotillin-1 localization in fraction 2 (the interface between 5 and 35% sucrose in the gradient) (Supplementary Material, Fig. S2), suggesting lipid rafts are enriched in fraction 2, which was used for subsequent analysis for the effect of treatments on protein levels in lipid rafts. In addition, as part of the characterization, proteins involved in the amyloidogenic pathway of APP processing were immunoblotted from each fraction and findings demonstrated the localization of these proteins in fraction 2 (Supplementary Material, Fig. S3), except α-secretase and β-CTF (Supplementary Material, Fig. S4). Based on the results, only pramlintide increased APP in lipid rafts significantly when compared to vehicle treated groups (*p* < 0.05) (Fig. 3a). Consistent with total brain homogenate results, BACE1 level in the lipid raft was not altered by amylin or pramlintide (Fig. 3a). However, pramlintide and amylin treated mice showed a significant increase in lipid raft levels of PSEN1, PSEN2, nicastrin and PEN2 subunits compared to control (*p* < 0.05; Fig. 3b). PSEN1, PSEN2 and nicastrin results differed from total brain homogenate.

### Amylin and pramlintide modulated GM1, GM2 and B4GALNT1 in total homogenate and/or lipid rafts

**Figure 4:**
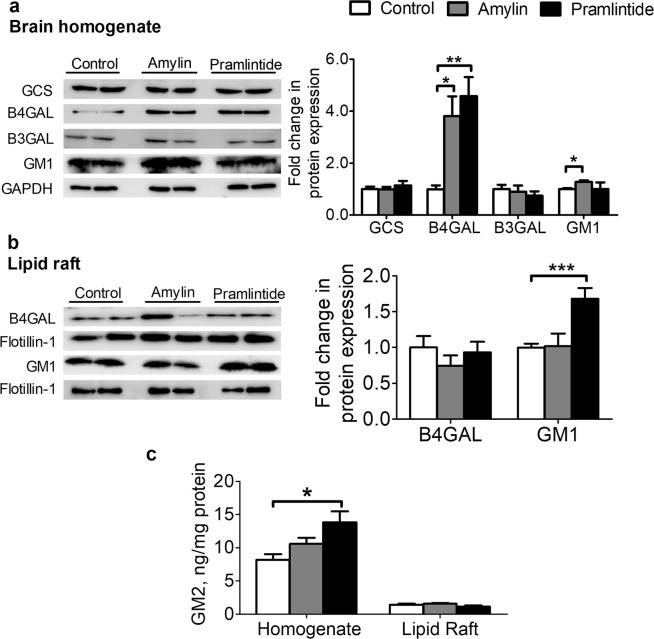
Effect of amylin and pramlintide effect on ganglioside production. **(a)** Representative Western blot and densitometry analysis of ganglioside demonstrated amylin and pramlintide did not alter the expression of GCS and B3GALT4; however, both amylin and pramlintide significantly increased the level of B4GALNT1 in total brain homogenate. Furthermore, amylin increased the level of GM1 compared to control group when measured from total brain homogenate. The blotted proteins were normalized to the level of GAPDH. **(b)** Representative Western blot and densitometry analysis of B4GALNT1 and GM1 in lipid rafts. Only pramlintide increased the level of GM1 in lipids rafts, while neither peptide altered the level of B4GALNT1 in lipid rafts. B4GALNT1 and GM1 were normalized to flotillin-1. The densitometry analysis is from n = 6 mice in each group, and western blot results are representative results from two different mice from each group. **(c)** Only pramlintide increased GM2 levels when measured from total brain homogenate; however, neither peptide altered GM2 levels in lipid rafts as determined by ELISA for n = 4 mice per group. Proteins were ran on different gels due to their close molecular weights size. Data were normalized to the total protein content from brain homogenate. Data is presented as mean ± SEM and the statistical significance for all result was assessed by student t-test, with *p < 0.05, **p < 0.01, ***p < 0.001 compared to control group.

Previous reports observed a role of GM1 gangliosides in modulating APP trafficking and processing^15^, and in increasing γ-secretase levels in lipid rafts^17^. Thus, in this study we evaluated amylin and pramlintide effects on gangliosides synthesis pathway including GM1, GM2, and the enzymes GCS, responsible for the biotransformation of ceramide into glucosylceramide, B4GALNT1 that converts GM3 to GM2, and B3GALT4 that converts GM2 to GM1 by addition of galactose^33^. Our results demonstrated amylin and pramlintide have no effect on GCS or B3GALT4 levels in total homogenate (Fig. 4a). However, compared to control, both amylin and pramlintide significantly increased B4GALNT1 levels in total brain homogenate (*p* < 0.05 and *p* < 0.001, respectively; Fig. 4a) but not in lipid rafts (Fig. 4b). GM2 was measured in total brain homogenate and lipid rafts using ELISA. Results showed that pramlintide, but not amylin, increased GM2 by 68% in total homogenate (*p* < 0.05; Fig. 4c). In contrast, neither amylin nor pramlintide altered GM2 levels in lipid rafts compared to control (Fig. 4c). GM1 levels in total brain homogenate and lipid rafts were also determined; findings from Western blot demonstrated amylin significantly increase GM1 in total brain homogenate (*p* < 0.05; Fig. 4a), while pramlintide significantly increased GM1 levels in lipid rafts (*p* < 0.001; Fig. 4b). In addition to the synthetic pathway for GM2 and GM1 gangliosides, their lysosomal degradative pathway was evaluated by measuring the activity of specific lysosomal enzymes responsible for their hydrolysis. Amylin and pramlintide did not alter the lysosomal enzyme activities as shown in Table 1.

**Table 1:** Lysosomal enzyme specific activity in mice brain tissues.

Specific activity				
Treatment	HexA	Hex Total	β-gal	α-Man
Control	173.7 ± 7.3	1601 ± 79.4	71.18 ± 6.3	1.975 ± 0.307
Amylin	1.98 ± 0.31	1747 ± 44.8	76.83 ± 3.6	1.867 ± 0.240
Pramlintide	183.5 ± 6.9	1821 ± 72.7	72.90 ± 4.2	2.750 ± 0.050

Specific activity is expressed as mean ±SEM for the nmol of 4-methylumbelliferone/mg protein per h at 37 °C. Average values were calculated from n = 4 mice from each treatment group. [HexA: A isozyme (αβ) of hexosaminidase; Hex Total: total hexosaminidase activity; β-gal: lysosomal β-galactosidase; α-Man: α-mannosidase].

### Amylin and pramlintide increased Aβ-related pathology

Both amylin and pramlintide bind to amylin receptor, which is a heterodimer of calcitonin receptor and receptor activity modifying protein 3 (CTR-RAMP3)^34^. To evaluate the effect of daily treatment of either peptide for 30 days on amylin receptor, RAMP3 was analyzed by Western blot in brain homogenate lysate and lipid rafts. While RAMP3 expression could not be detected in lipid rafts, neither peptide altered RAMP3 levels detected as monomer, homodimer or heterodimer when compared to control group (Supplementary Material, Fig. S5).

**Figure 5:**
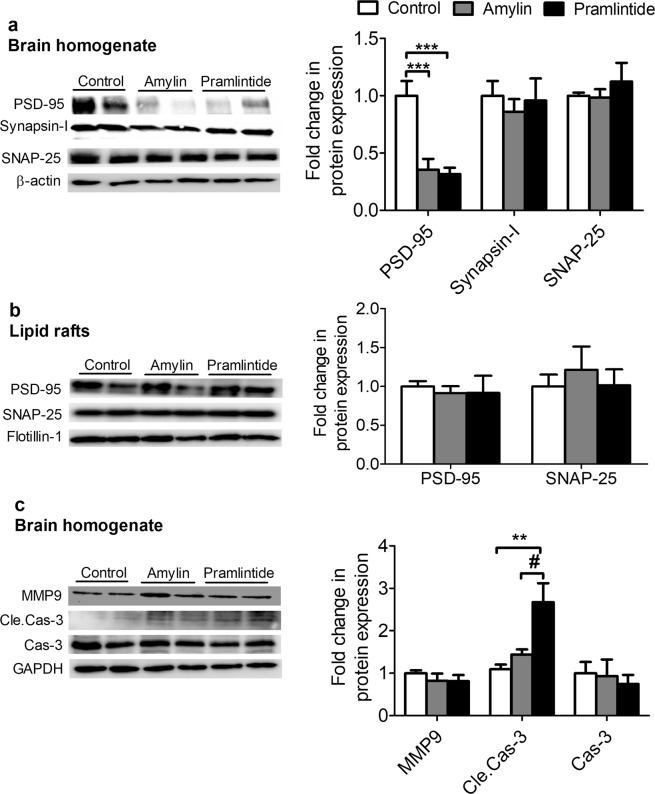
Effect of amylin and pramlintide on Aβ-related pathology. **(a)** Representative Western blot and densitometry analysis of synaptic markers in mice brain homogenates showed amylin and pramlintide significantly reduced the level of PSD-95, without affecting SNAP-25 and synapsin-1 in total brain homogenate. Data were normalized to β-actin. **(b)** Representative Western blot and densitometry analysis of synaptic markers and LRP1 in lipid rafts. Amylin and pramlintide had no effect on PSD-95 and SNAP-25 levels in lipid rafts; however, both peptides decreased the level of LRP1. All proteins from lipid rafts were normalized to flotillin-1. **(c)** A representative Western blot and densitometry analysis demonstrated that amylin and pramlintide significantly increased cleaved caspase-3 (Cle.Cas-3) compared to amylin and control group without affecting the levels of total caspase-3 (Cas-3) and MMP9. MMP9 was ran on different gel due to molecular weight similarity. All proteins were normalized to their corresponding housekeeping proteins. The densitometry analysis is from n = 6 mice in each group. The western blot results are representative results from two different mice from each group. Data is presented as mean ± SEM and the statistical significance for all result was assessed by student test, with *p < 0.05, **p < 0.01, and ***p < 0.001.

The effect of amylin and pramlintide on pre-synaptic markers SNAP-25 and synapsin-1 and post-synaptic marker, PSD-95, were also evaluated in mice brain homogenate by Western blotting. Both amylin and pramlintide significantly reduced PSD-95 expression (*p* < 0.01 and *p* < 0.001, respectively), without altering SNAP-25 or synapsin-1 levels (Fig. 5a). When tested in lipid rafts, neither peptide altered the levels of PSD-95 nor SNAP-25 compared to control group (Fig. 5b). LRP1 was also determined in lipid rafts where both treatments reduced its level significantly (*p* < 0.05, Fig. 5b). The effect of treatments on the apoptotic marker cleaved caspase-3 was also evaluated in brain homogenate, and the results showed amylin and pramlintide significantly increased cleaved caspase-3 levels in mice brains (*p* < 0.01), without altering total caspase 3 (Fig. 5c). Moreover, neither peptide altered the matrix metalloproteinase MMP9 level when compared to control group (Fig. 5c).

**Figure 6:**
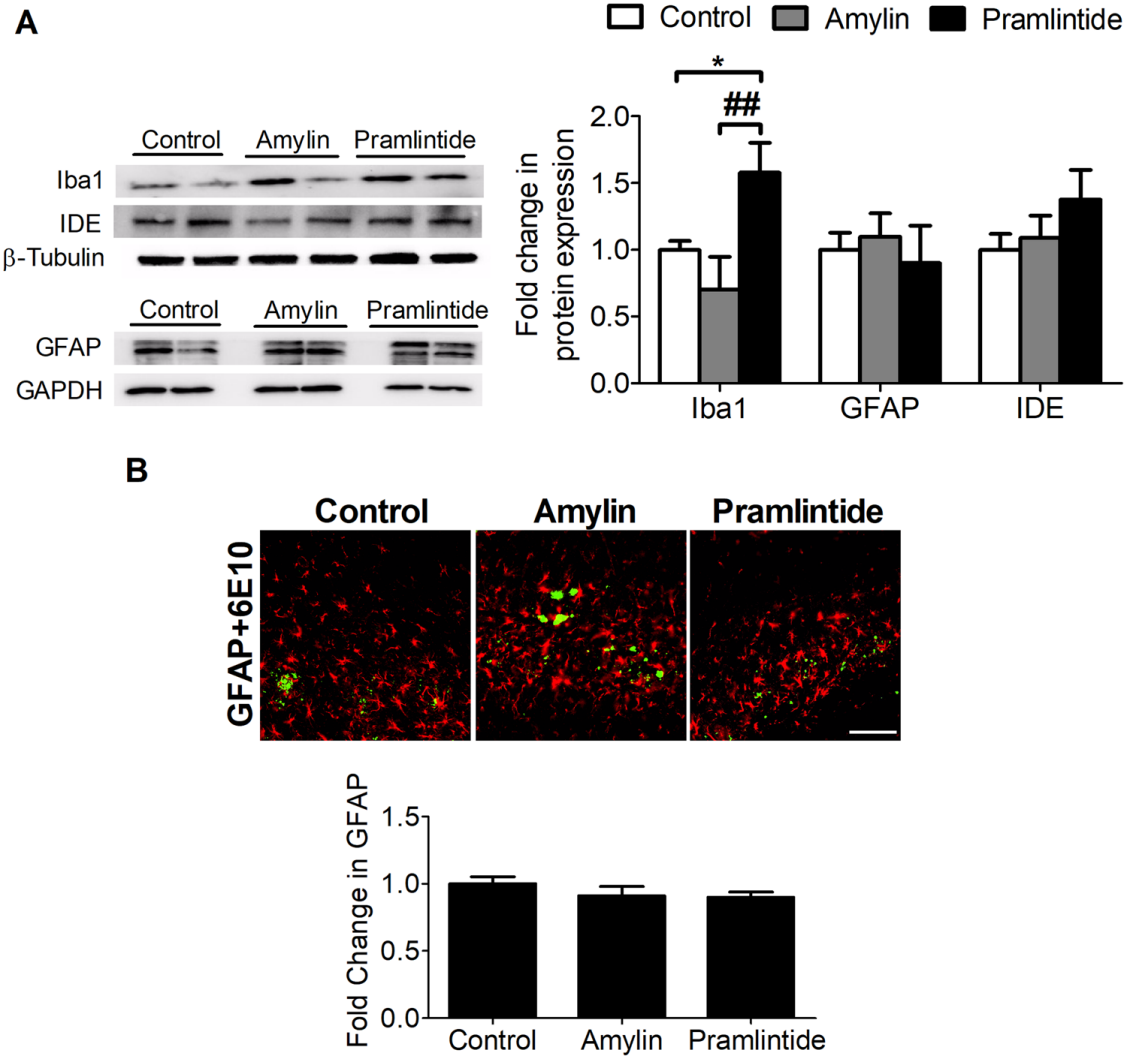
Effect of amylin and pramlintide on neuroinflammation markers and IDE. **(A)** Representative Western blot and densitometry analysis of glial cells markers in mice brain homogenates showed pramlintide significantly increased the level of Iba1 compared to control and amylin. Neither treatment altered GFAP or IDE levels in total brain homogenate. The densitometry analysis is from n = 6 mice in each group. The western blot results are representative results from two different mice from each group. Proteins were ran on different gels. All proteins were normalized to their corresponding housekeeping proteins. **(B)** Immunohistochemical images and analysis of GFAP (red) in brain hippocampus showed the treatments had no effect on GFAP intensity and astrocytes shape. The quantification of GFAP intensity was assessed from n = 3 in each group. Total Aβ was immunostained with 6E10 (green). Scale bar = 100 µm. Data is presented as mean ± SEM and the statistical significance for all result was assessed by student t-test, with *p < 0.05 compared to control group; ^##^*p* < 0.01 com*p*ared to pramlintide.

Aβ is cleaved by degrading enzymes such as IDE^35^, whose level is altered in T2D and AD^36^. In this study, treatment with amylin or pramlintide had no significant effect on IDE level compared to control measured from total brain homogenate (Fig. 6A). Neuroinflammation is another hallmark of AD, and increased brain Aβ levels is associated with microglia activation and astrogliosis that produce an inflammatory cascade leading to neuronal toxicity and neurodegeneration^37^. Treatment effects on glial activation markers were evaluated by immunostaining and Western blotting. Pramlintide significantly increased Iba1, a microglia marker when compared to control and amylin (*p* < 0.05 and *p* < 0.01, respectively) (Fig. 6A). However, neither peptide modulated astrogliosis as determined by Western bolt and immunostaining of astrocytes marker, glial fibrillary acidic protein (GFAP) in terms of intensity or morphology (Fig. 6A,B).

## Discussion

Amylin is a gut–brain axis hormone which crosses the BBB^38^ and exert its effect on the CNS^39^. Pramlintide is amylin’s clinically available analog, which was developed by substituting prolines at positions 25, 28, and 29 of human amylin to prevent amylin oligomerization or aggregation^40^. Amylin shares similar secondary structure with Aβ^25^, thus Aβ binds amylin receptor as well^26^. However, the intracellular signaling is different between the two ligands amylin and Aβ^40^. Using different AD animal models, multiple studies have shown that amylin ameliorates AD pathology by decreasing neuroinflammation and increasing Aβ clearance from brain to blood^30–32,41^. On the other hand, a number of studies has shown that amylin is involved in the pathogenesis of AD by inducing neuroinflammation and apoptosis^25,28,42–46^. Findings from our study agree with the latter studies where amylin and pramlintide increased Aβ production and exacerbated Aβ-related pathology in TgSwDI mice brains, however, without worsening memory as assessed by MWM. TgSwDI mouse model expresses human APP harboring the Swedish, Dutch and Iowa mutations and it is characterized by early (2 to 3 months of age) and aggressive Aβ accumulation on the wall of blood vessels and increased Aβ_40_ production^47^.

Our data suggest a previously undisclosed link between APP processing and amylin or pramlintide^28,30–32^. The increased level of amyloidogenic pathway proteins in lipid rafts, and to a lower extent in total brain homogenate, caused by amylin and pramlintide signifies the importance of evaluating APP processing at the plasma membrane level. Amylin and pramlintide increased the expression of γ-secretase 4-subunits in lipid rafts, an effect that was not fully observed in total homogenate. The increased level of γ-secretase complex subunits in lipid rafts might be responsible for the increased Aβ burden as confirmed by our ELISA and immunohistochemistry results, which are consistent with other studied demonstrating APP processing in lipid rafts^10,11^. On the other hand, neither peptide altered BACE1 expression in brain homogenates and lipid rafts, suggesting the observed increase in sAPP-β by pramlintide is due to increased APP trafficking to the lipid raft.

**Figure 7:**
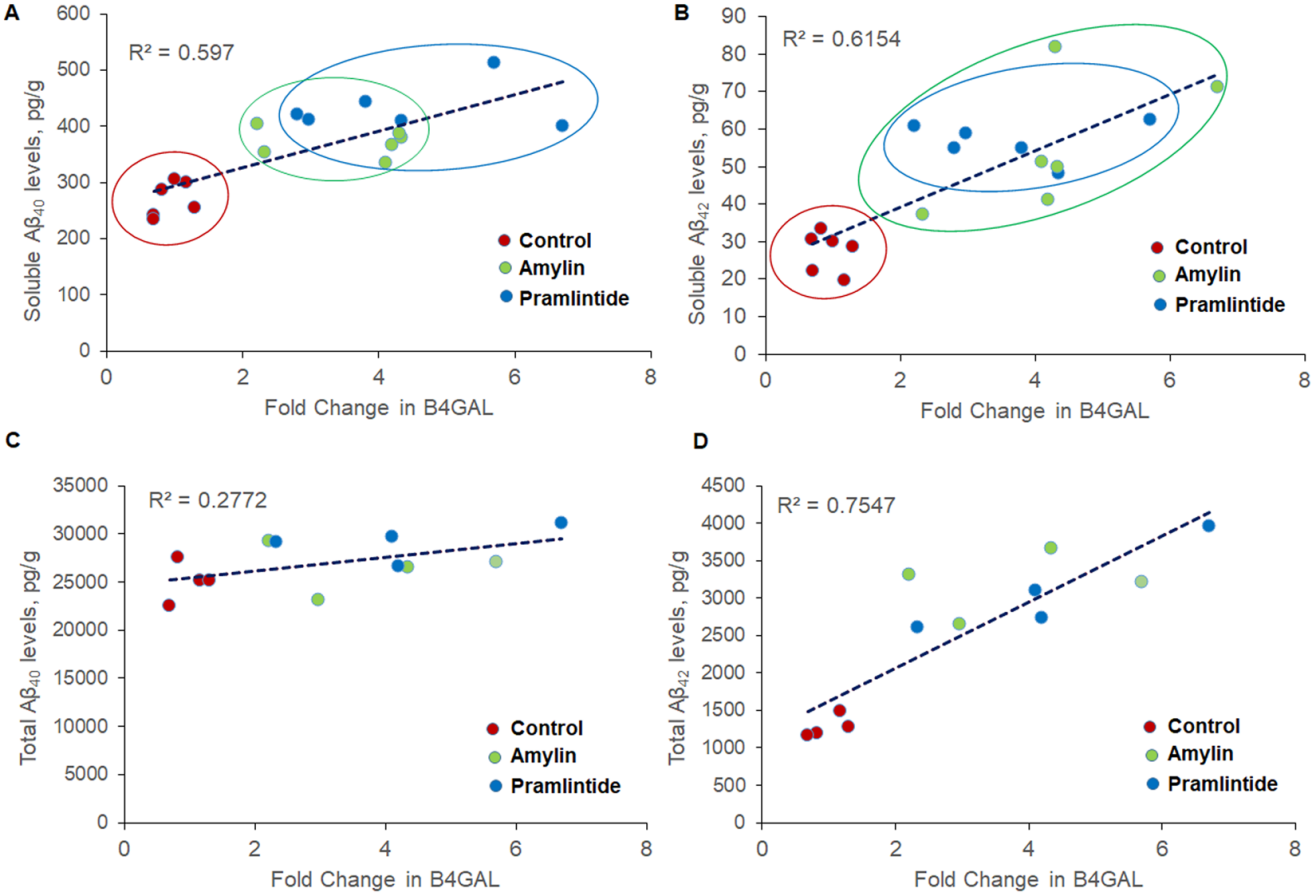
Correlation analysis between fold change in B4GALNT1 and Aβ levels revealed positive correlation between B4GALNT1 and soluble Aβ_40_ **(A)** and soluble Aβ_42_ **(B)**, and B4GALNT1 and total Aβ_42_ **(D)**. The correlation between B4GALNT1 and total Aβ_40_ **(C)**, on the other hand, was small as demonstrated by low coefficient of determination (R^2^). For **A** and **B**, n = 6 mice/treatment group were used; and for **C** and **D**, n = 4 mice/treatment group were used.

To explain the observed effect of amylin and pramlintide on the amyloidogenic processing of APP in lipid rafts, the effect of both peptides on the synthesis of GM1 and GM2 gangliosides was evaluated. Several studies have reported that GM1 and GM2 are involved in AD pathology^14,33,48^, and changes in brain ganglioside composition were observed in patients with AD^16,49^, implicating a direct association of gangliosides with AD. GM1 is the most abundant ganglioside in the brain and it binds to Aβ at the cell surface, which accelerates its extracellular deposition^14^. Furthermore, available studies reported reduced synthesis of GM1 is associated with decreased transport of APP to cell surface^16^, and that treatment of neuronal and non-neuronal cells with GM1 increased Aβ_40/42_ secretion by affecting the activity of γ-secretase^17^. In a recent study, Yamaguchi and colleagues reported that SK-MEL-28-N1 cells treatment with GM2 and GM1 demonstrated higher levels of APP and BACE1 compared to GM3 treated cells, and that increased levels of B4GALNT1 increased APP trafficking and localization in lipid rafts^33^. Similarly, our data from western blotting analysis revealed both amylin and pramlintide increased B4GALNT1 in total homogenate, and GM1 levels in total homogenate (amylin) and lipid rafts (pramlintide), suggesting a role in increased APP processing and Aβ production. Beside Western blot data, an additional experiment was performed to visualize co-localization of APP with GM1-enriched membrane lipid rafts (stained by Cholera Toxin Subunit B- Alexa Fluor 594 conjugate) by fluorescence staining, and results showed higher co-localization of APP with GM1-enriched membrane lipid rafts in pramlintide treated mice when compared to other treatment groups (Supplementary Material, Fig. S6), supporting Western blot findings. Furthermore, correlation analysis between fold change in B4GALNT1 and Aβ levels revealed positive correlation between B4GALNT1 and soluble Aβ_40_ and Aβ_42_, and B4GALNT1 and total Aβ_42_ (Fig. 7). These results suggest that B4GALNT1 directly influenced Aβ levels where increased levels of B4GALLNT1 by amylin and pramlintide was associated with increased levels of Aβ. Further analysis of other gangliosides demonstrated only pramlintide increased GM2 levels when measured in total brain homogenate without altering its effect in lipid rafts. To explain the increased levels of GM1 caused by amylin, B3GALT4, the enzyme responsible for GM1 synthesis from GM2, was analyzed and results showed neither amylin nor pramlintide altered this enzyme. Next, and as the increased level of GM1 and GM2 could also be explained by alteration in their lysosomal degradation, the activity of β-gal which cleaves GM1 to GM2, and HexA which cleaves GM2 to GM3, were evaluated. However, data showed no significant alteration in lysosomal enzyme activities. Collectively, while further investigation is necessary, our findings suggest increased GM1 levels could be explained indirectly by increased B4GALNT1, which increased GM2 ganglioside, the precursor of GM1. However, GM2 increase was only observed with pramlintide.

Amylin and pramlintide significantly reduced sAPP-α, a finding that is consistent with previously reported studies demonstrated that SH-SY5Y-APP695 treatment with GM1 significantly decreased sAPP-α^17^. Stiffening of the membrane due to a high level of GM1 may decrease sAPP-α by limiting lateral movement and required contact between α-secretase enzyme and substrate^17^. To confirm the effect of amylin and pramlintide on α-secretase, the enzyme level was determined by Western blot and results showed that neither peptide altered α-secretase level in brain homogenate; on the other hand, we were not able to detect the enzyme in fraction 2 in lipid rafts (Supplementary Material, Fig. S4).The interaction with GM1 has been reported as an important factor in mediating aggregation and toxicity of Aβ and amylin^50,51^. In addition, amylin interaction with plasma membrane is thought to be the main factor determining the death of pancreatic β-cells in T2D^52^, where several *in vitro* studies reported seeding and aggregation of Aβ and amylin on synthetic membrane are enhanced by GM1^53,54^.

Increased accumulation of Aβ due to its increased production by amylin and pramlintide could lead to synaptic loss and microglial activation as demonstrated by increased Iba-1, increased apoptotic marker cleaved caspase-3 and reduced post-synaptic marker PSD-95. Increased brain Aβ is expected to activate glial cells and produce inflammatory cascade^33^. This effect contradicts previously reported neuroprotective effect of amylin against neuroinflammation where amylin reduced Iba1, CD68, and pro-inflammatory cytokines^30,31^. The reduction in total PSD-95 expression following amylin and pramlintide treatments was associated with reduced LRP1 in lipid rafts fraction, but not in total homogenate. In neuronal cells, LRP1 partitions between both lipid rafts and non-raft membrane fractions^55^, and it’s signaling activation leads to neurite outgrowth and cell growth^56^. LRP1 interacts with the active pool of PSD-95 and reduction in total PSD-95 is expected to reduce total LRP1 in neuronal cells^57^. It has also been reported that localization of LRP1 to lipid rafts reflects the activity of PSD-95, which is known to cluster other membrane proteins in rafts through its scaffolding activity^58^. Therefore, the reduction in total PSD-95 level due to amylin and pramlintide could explain the reduction in LRP1in lipid rafts. On the other hand, available studies, unlike our findings, reported pramlintide treatment for 5 weeks increased expression of the presynaptic marker synapsin 1^41^. In this study, the authors used SAMP8 mice at the age of 6 months, and this mouse model exhibits age-related dementia. However, whether similar effect will be observed with pramlintide under pathological insult requires further investigation.

Increased parenchymal Aβ levels and related pathology in the brains of mice treated with amylin and pramlintide for 30 days, however, was not translated to impairment in memory function when compared to vehicle treated mice as determined by MWM behavioral studies. While additional studies are necessary to explain this observation, we speculate that a further decline in memory performance could be observed with the chronic treatment for longer time (i.e. more than 30 days) associated with exacerbated pathology consequent to increased Aβ production in TgSwDI mice brains. Furthermore, while studies with pramlintide are limited in the literature, available studies with amylin show contradicting effects against Aβ-related pathology as well as memory function in AD mouse models^31,32,41,45^. An explanation(s) for this discrepancy is not clear, however, the mouse model used in our study is different from others. In this study we used the CAA/AD model TgSwDI, which is characterized by Aβ deposition not only in the parenchyma but also on brain microvessels. Though we selected a dose and route of administration shown to be protective^30–32^, the opposite effect was observed. In a review by Qiu *et al*., the authors explained the discrepancy observed with amylin could be aggregation dependent^40^. For example, treatment of rat cortical neurons with *human* amylin at 50 µM concentration caused neurotoxicity due to amylin aggregation, whereas at the same concentration, *rat* amylin did not show aggregation or neurotoxicity^59^. Also, at lower concentrations (2.5 nM – 2.5 μM), *human* amylin was able to antagonize aggregated Aβ_42_-induced neurotoxicity^40^. In addition, low vs. high concentrations of amylin could activate different receptors based on the degree of amylin aggregation^44^. In this scenario, the neuroprotective effect of non-aggregated amylin is based on binding a different receptor than that bound by aggregated amylin^60^. Thus, to better understand and clarify amylin and pramlintide effects against AD, dose despondent studies are necessary. Furthermore, additional experiments that would be important to perform include studies in wild type mice as well as in female TgSwDI mice to understand treatments effect in the absence of pathology and whether this effect is sex dependent.

In conclusion, findings from our study suggest that amylin and pramlintide have the potential to increase Aβ-related pathology through modulating γ-secretase activity and APP processing in lipid rafts, and by increasing GM1 ganglioside levels.

## Methods

### Reagents and antibodies

Synthetic human amylin was purchased from Anaspec (Cat# AS-60254-1), and Pramlintide was purchased from Biotang Inc. (Cat# BT-HOR-300), Brij®98 was purchased from Thermo Fisher Scientific, thioflavin-S was from Sigma Aldrich and NP-40 lysis buffer was purchased from Alfa Aesar. All other chemicals were purchased from VWR. The following primary antibodies were used to probe the membranes in immunoblotting: anti-human sAPP-β and anti-human sAPP-α (Immuno-Biological Laboratories Co., Ltd); anti-APP A4 antibody clone 22C11 and anti-APP-C99 antibody clone M (Millipore); BACE1, LRP1, glucosylceramide synthase (GCS), B4GALNT1, and Iba1 antibodies (abcam); presenilin 1, presenilin 2, nicastrin, PEN2, caspase-3 and synapsin-1 antibodies (Cell Signaling); SNAP-25, zona-occludin 1 (ZO1), occludin, GAPDH, matrix metallopeptidase 9 (MMP9), B3GALT4, Cholera Toxin Subunit B (Recombinant)-HRP, and flotillin 1 antibodies (Invitrogen); IDE, RAMP3, ADAM10 (B-3) for α-secretase and β-tubulin antibodies (Santa Cruz Biotechnology); and PSD-65 antibody (GeneTex). The secondary antibodies used in immunoblotting are goat anti-rabbit IgG (H+L)-HRP and goat anti-mouse IgG (H+L)-HRP (Invitrogen) and goat IgG HRP-conjugated (R&D systems). The following antibodies were used in the immunohistochemistry experiments: rabbit polyclonal collagen IV antibody (Millipore), donkey polyclonal Alexa Flour 647 antibody to rabbit IgG (abcam), Alexa Fluor-488 conjugated anti-Aβ antibody (6E10) (Biolegend), and rabbit GFAP antibody (Santa Cruz Biotechnology).

### Animal treatment

All animal experiments and procedures were approved by the Institutional Animal Care and Use Committee of the University of Louisiana at Monroe and according to the National Institutes of Health guidelines, as in Principles of Laboratory Animal Care (NIH publication No. 86-23, revised 1996). Males TgSwDI transgenic mice (Jackson Laboratories) at age of 4 months were housed in plastic cages under standard conditions, 12-h light/dark cycle, 22 °C, 35% relative humidity, and *ad libitum* access to water and food. Mice were treated with i.p. injections of human amylin (200 µg/kg/day; n = 8), pramlintide (200 µg/kg/day; n = 8), or PBS as vehicle (n = 8) for 30 days. At the end of treatment period, mice were deeply anesthetized with ketamine (100 mg/kg)/xylazine (12.5 mg/kg) cocktail i.p., then decapitated for brains collection. Effect of peptides on blood glucose levels was assessed on final day of treatment without significant difference and the readings were 150.7 ± 1, 151.3 ± 0.9 and 151.5 ± 1.5 mg/dl for vehicle, amylin and pramlintide treated mice, respectively (*p* > 0.05).

### Behavioral testing

The Morris water maze (MWM) test was performed for TgSwDI mice to assess learning and memory performance at the end of the treatment using protocols similar to those described previously^61^. All mice underwent training 3 times a day for 4 consecutive days. The platform was kept at the same quadrant during the entire course of the experiment. Mice were required to find the hidden platform utilizing the distal spatial cues available in the room. Conditions were maintained the same during all the experiments. An overhead camera connected to a computerized tracking system (SMART 3.0 Platform, Panlab Harvard apparatus (Holliston, MA)) was used to record the movements of the mice. The results including swimming speed, latency to target, swimming distance, and number of entries in target quadrant were collected and used for analysis.

### Analysis of Aβ burden in mice brains

Brain weights were measured and homogenized in two volumes of DPBS (137 mM NaCl, 8.1 mM Na_2_HPO_4_, 2.7 mM KCl, 0.9 mM CaCl_2_, 5 mM D-glucose, 0.5 mM MgCl_2_, 1.46 mM KH_2_PO_4_, 1 mM Na-pyruvate) to prepare brain homogenate. Homogenate samples were lysed (1:1.5) with NP-40 lysis buffer containing protease arrest on ice for 45 min. From this homogenate, 100 µl were centrifuged at 20,817 *x g* for 15 min at 4 °C to collect supernatant/lysate that contain soluble Aβ. To measure oligomeric and insoluble Aβ from total brain homogenate, a 2-step serial extraction procedure was used as described previously with modification^62^. In brief, the remained pellet following soluble Aβ extraction was mixed with 2% SDS in PBS containing protease arrest with homogenization, followed by sonication for 10 min and centrifugation at 20,817 *x g* for 60 min at 22 °C. The supernatant was collected and stored in −80 °C. To isolate insoluble Aβ, the pellet from the second fractionation was re-suspended in 70% formic acid in PBS containing protease arrest, followed by homogenization and sonication for 10 min, and finally centrifugation at 20,817 *x g* for 60 min at 4 °C. In addition, to measure total Aβ_40_ and Aβ_42_, 70% formic acid was added to brain homogenate, followed by homogenization, sonication, and centrifugation as described above. Supernatant was collected and stored in −80 °C. The formic acid fraction was neutralized 1:20 with 1 M Tris/0.5 M Na_2_HPO_4_. The soluble, oligomeric and insoluble Aβ_40_ and Aβ_42_ were measured separately by two commercial ELISA kits (Thermo Fisher Scientific) for Aβ_40_ and Aβ_42_.

### Fractionation of lipid rafts

Lipid rafts fractionation was performed as reported previously with modification^63^. Eighty microlitters from each brain homogenate in DPBS was mixed with 600 µl of 1% Brij^®^98, 25 mM Tris-HCl pH 7.5, 150 mM NaCl, 5 mM EDTA, 1 mM PMSF, and then incubated on ice for 30 min. The suspension was centrifuged at 1,000 *x g* for 5 min at 4 °C. Five hundred microlitter from sample supernatant was mixed with equal volume of 80% (wt/vol) sucrose in TNE buffer (25 mM Tris-HCl pH 7.5, 150 mM NaCl, 5 mM EDTA) and placed at the bottom of an ultracentrifuge tube. Then, 4 ml discontinuous sucrose gradients in TNE buffer consisting of 3 ml 35% (wt/vol) sucrose and 1 ml 5% (wt/vol) sucrose were overlayered on the top. The sucrose gradient was centrifuged at 260,000 *x g* for 3 h at 4 °C using Beckman Coulter ultracentrifuge in SW55 Ti rotor. The fractions (500 µl each) from each sample were collected from top to bottom of the tube and then stored in −80 °C until analysis.

### Western blot analysis

Brain homogenates in DPBS were lysed on ice for 45 min with NP-40 lysis buffer containing protease arrest and then centrifuged at 20,817 *x g* for 15 min at 4 °C, followed by collecting and storing the supernatant for immunoblotting. Total protein content was measured by Pierce™ BCA Protein Assay kit (Thermo Fisher Scientific). Equal amounts of protein (20 µg) from brain homogenate lysates and lipid raft fractions were subjected to SDS-PAGE followed by immunoblot analysis according to a standard procedure. To detect proteins, 12% Tris-glycine polyacrylamide gels were used. For GM1 detection, 15% gels for GM1 separation were used, membranes were then blocked and incubated with Cholera Toxin Subunit B (recombinant)-HRP for 1 h at room temperature with shaking followed by imaging. For amylin receptor (RAMP3) immunoblotting, we used commericially avialable stain free kit (Bio-Rad). Blots were developed using a chemiluminescence detection kit (SuperSignal West Femto substrate; Thermo Fisher Scientific); bands were visualized by ChemiDoc imaging system (Bio-Rad) and then analyzed by Image Lab software v 6.0 (Bio-Rad). The results were expressed as fold change in protein level compared to control group after normalization to the house keeping proteins.

### Immunohistochemistry analysis

Brain sections of 16 μm-thick were prepared using Leica CM3050S Research Cryostat. All brains slices were methanol-fixed and blocked for 30 min with 10% normal donkey serum in PBS. For the detection of Aβ-plaques load, we followed a previously published protocol with slight modification^62^. Briefly, the sections were immuostained with rabbit polyclonal collagen IV antibody (1:200) to detect brain microvessels followed by donkey polyclonal Alexa Flour 647 antibody to rabbit IgG (1:200), which were then incubated in filtered 0.02% thioflavin-S (Thio-S) solution prepared in 70% ethanol for 30 min to detect Aβ deposits. Sections were then washed in 70% ethanol for 15 min and covered with cover-clips for imaging. For total Aβ load detection, brain slices were double immunostained for microvessels and Alexa Fluor-488 conjugated anti-Aβ antibody (6E10) (1:200). Double immunostaining of astrocytes and Aβ was performed using rabbit GFAP antibody (1:200), and for detection donkey polyclonal Alexa Flour 647 antibody to rabbit IgG (1:200) was used. For each treatment, image acquisition was performed in 10 tissue sections spanning the hippocampus, each separated by 150 μm (total of 40 sections per mouse). Images were captured using Nikon Eclipse Ti−2 inverted fluorescence microscope (Nikon). Quantification of all images was performed using NIS Element AR analysis v5 (Nikon) after adjusting for threshold.

### GM2 ganglioside analysis by ELISA

Brain homogenate in DPBS was diluted 1:5 with PBS and centrifuged at 950 *x g* for 20 min at 4 °C. GM2 was measured in the supernatant following the manfacturer protocol (MyBioSource, Cat # MBS017456). GM2 was also measured in lipid rafts and the level of GM2 from total brain homgenate and lipid rafts were normalized to protein content in total brain homogenate.

### Assay of lysosomal ezyme activities

The lysed brain homogenate in NP-40 lysis buffer was diluted 1:1 in citrate phosphate buffer and the lysosomal enzyme activities for beta-galactosidase (β-gal); hexosaminidase A (HexA), total hexosaminidase (A,B and S isozymes; Hex T), and alpha-mannosidase (α-Man) were measured with synthetic substrates based on released amount of 4-methylumbelliferone as previously described^64^. α-Man cleaves lysosomal substrates outside the the gangliosides pathway and it is used as assay control.

### Statistical analysis

All values were expressed as mean ± SEM. Statistical analysis was done with Prism v5.0 software (Graphpad). The statistical significance for all result was assessed by Student t-test. A *p* value of <0.05 was considered statistically significant.

## Supplementary information

Supplementary information

## Data Availability

The data that support the findings of this study are available from the corresponding author, upon reasonable request.
