# Author Correction: Amylin and pramlintide modulate γ-secretase level and APP processing in lipid rafts

**DOI:** 10.1038/s41598-020-68281-y

**Published:** 2020-07-01

**Authors:** Youssef M. Mousa, Ihab M. Abdallah, Misako Hwang, Douglas R. Martin, Amal Kaddoumi

**Affiliations:** 1 Department of Drug Discovery and Development, Harrison School of Pharmacy, Auburn University, Auburn, USA; 2 Scott-Ritchey Research Center, Auburn University, Auburn, USA; 3 Department of Anatomy, Physiology and Pharmacology, College of Veterinary Medicine, Auburn University, Auburn, USA; 4 Center for Neuroscience Initiative, Auburn University, Auburn, AL USA

Correction to: *Scientific Reports* 10.1038/s41598-020-60664-5, published online 28 February 2020

This Article contains errors.

In Figure 2, Figure 2d is missing, which shows fold change in β-CTF protein expression. The correct Figure 2 appears below as Fig. 1.

**Figure 1:**
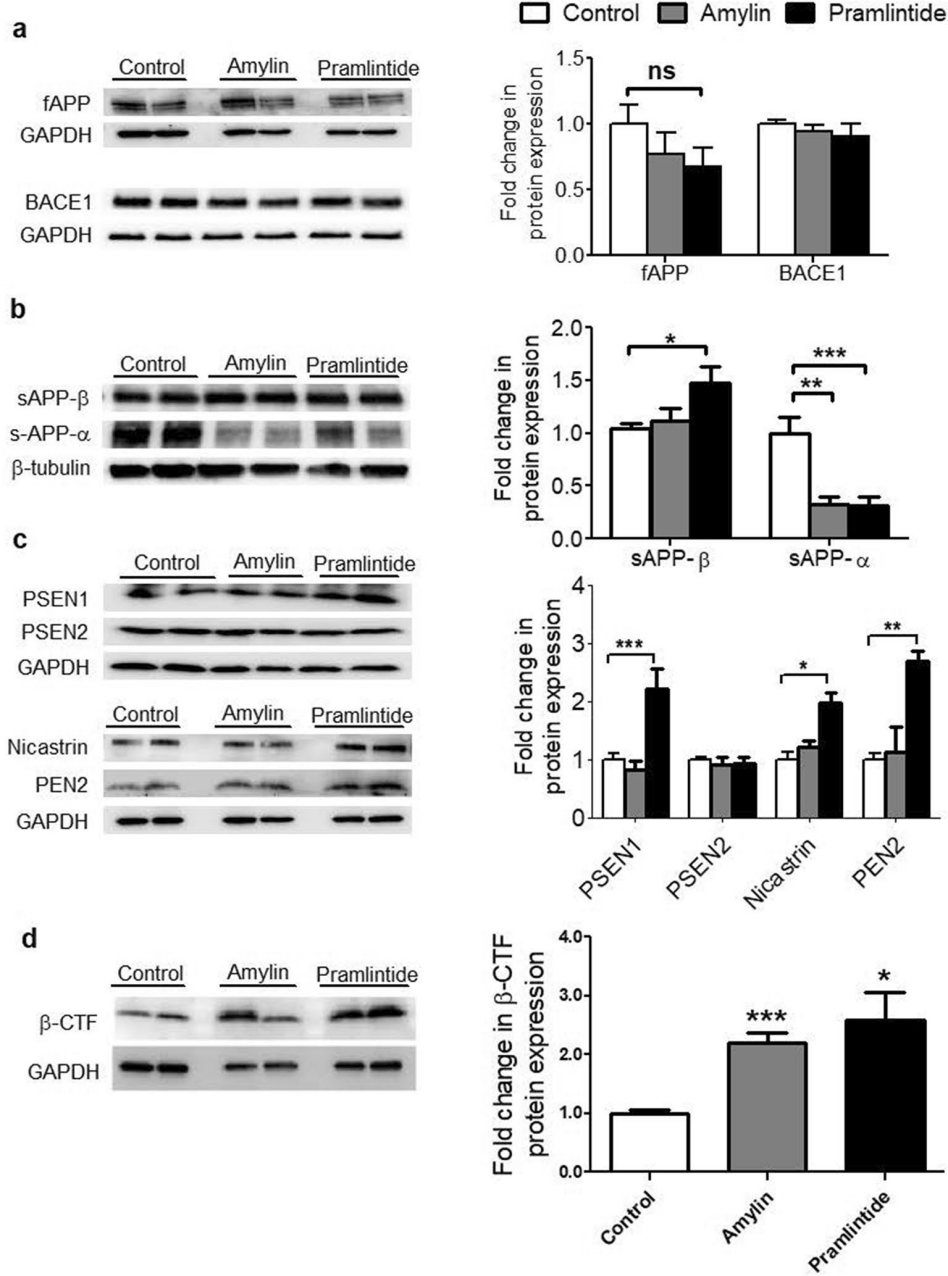
Effect of amylin and pramlintide on APP processing in total brain homogenate. (**a**) Representative Western blot and densitometry analysis of full-length APP (fAPP) and BACE1 demonstrated amylin and pramlintide did not alter full-length APP (fAPP) and BACE1 in mice brain homogenates. The fAPP and BACE1 levels were normalized to GAPDH level. (**b**) Representative Western blot and densitometry analysis of sAPP-β and sAPP-α in mice brains demonstrated pramlintide significantly increased sAPP-β compared to control, whereas reduction in the level of sAPP-α was observed after treatment with amylin and pramlintide. The levels of sAPP-β and sAPP-α were normalized to the level of β-tubulin. sAPP-β and sAPP-α were ran on different gels due to molecular weight similarity. (**c**) Representative Western blot and densitometry analysis of γ-secretase subunits in mice brains demonstrated pramlintide caused a significant increase in PEN2 subunit when compared to control and amylin; however, neither peptide influenced the other γ-secretase subunits PSEN1, PSEN2 and nicastrin. The level of γ-secretase subunits was normalized to level of GAPDH in each corresponding lane. PSEN1 and PSEN2 were ran on different gels due to molecular weight similarity. (**d**) Representative Western blot and densitometry analysis of β-CTF in mice brains demonstrated amylin and pramlintide caused a significant increase in β-CTF when compared to control. Data is presented as mean ± SEM, and the densitometry analysis is from n = 6 mice in each group. The western blot results are representative results from two different mice from each group. Data is presented as mean ± SEM and the statistical significance for all result was assessed by student t-test, with *p < 0.05, **p < 0.01, ***p < 0.001 compared to control group; ^#^*p* < 0.05 compared to pramlintide, and ns = not significant.

In Figure 5b, which shows fold change in protein expression in lipid rafts, ‘LRP1’ is not represented. The correct Figure 5 appears below as Fig. 2.

**Figure 2:**
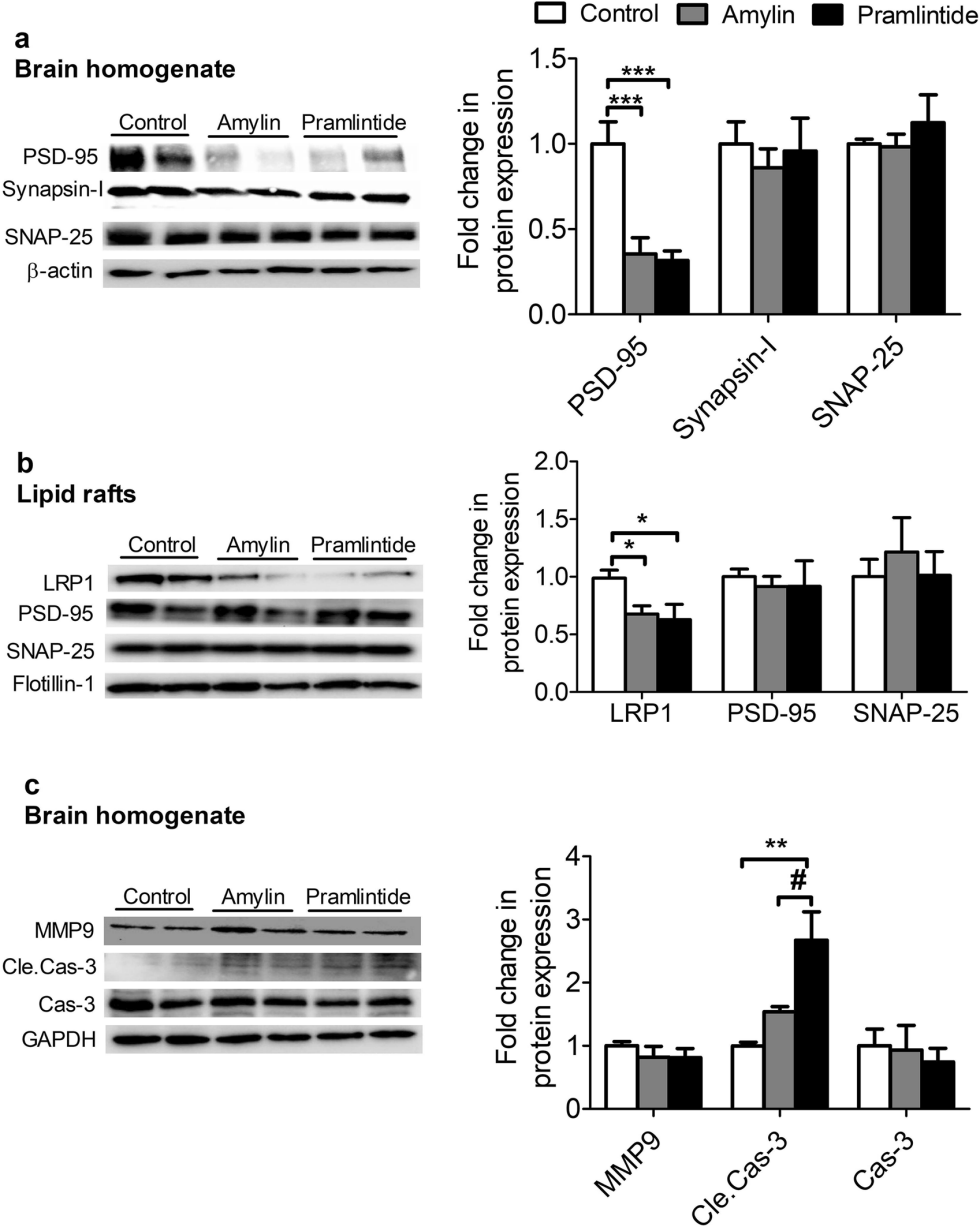
Effect of amylin and pramlintide on Aβ-related pathology. (**a**) Representative Western blot and densitometry analysis of synaptic markers in mice brain homogenates showed amylin and pramlintide significantly reduced the level of PSD-95, without affecting SNAP-25 and synapsin-1 in total brain homogenate. Data were normalized to β-actin. (**b**) Representative Western blot and densitometry analysis of synaptic markers and LRP1 in lipid rafts. Amylin and pramlintide had no effect on PSD-95 and SNAP-25 levels in lipid rafts; however, both peptides decreased the level of LRP1. All proteins from lipid rafts were normalized to flotillin-1. (**c**) A representative Western blot and densitometry analysis demonstrated that amylin and pramlintide significantly increased cleaved caspase-3 (Cle.Cas-3) compared to amylin and control group without affecting the levels of total caspase-3 (Cas-3) and MMP9. MMP9 was ran on different gel due to molecular weight similarity. All proteins were normalized to their corresponding housekeeping proteins. The densitometry analysis is from n = 6 mice in each group. The western blot results are representative results from two different mice from each group. Data is presented as mean ± SEM and the statistical significance for all result was assessed by student test, with *p < 0.05, **p < 0.01, and ***p < 0.001.

