# Supplementary material for: Amylin and pramlintide modulate γ-secretase level and APP processing in lipid rafts
Source: Sci Rep. 2020 Feb 28;10:3751. doi: 10.1038/s41598-020-60664-5 (PMC7048857; doi:10.1038/s41598-020-60664-5)

Amylin and pramlintide modulate γ-secretase level and APP processing in lipid rafts

Youssef M. Mousa,[1] Ihab M. Abdallah,[1] Misako Hwang,[2] Douglas R. Martin,[2,3,4] Amal Kaddoumi[1,4*]

[1] Department of Drug Discovery and Development, Harrison School of Pharmacy, Auburn University.

[2] Scott-Ritchey Research Center, Auburn University

[3] Department of Anatomy, Physiology and Pharmacology, College of Veterinary Medicine, Auburn University.

[4] Center for Neuroscience Initiative, Auburn University, Auburn, AL

Supplementary Fig. S1. The effect of amylin or pramlintide treatment on (**A**) swimming speed, (**B**) latency to target, (**C**) swimming distance, and (**D**) number of entries in target quadrant in TgSwDI mice. Amylin or pramlintide did not alter memory function compared to vehicle treated mice. Data is presented as mean ± SEM from n=4 mice in each group. For statistical significance, Student t-test was used; ns=not significant.

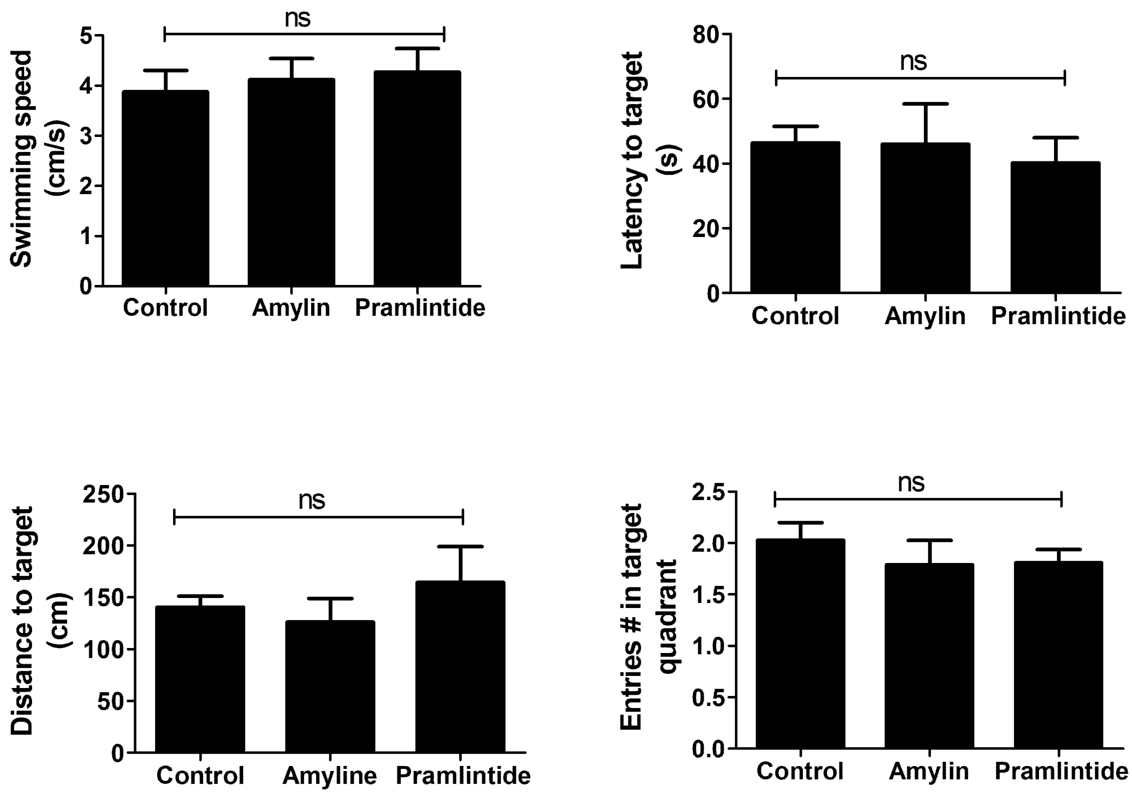

Supplementary Fig. S2. The separation of lipid rafts using discontinuous sucrose gradient and ultracentrifugation. Lipid rafts were found in fraction 2 and the non-raft fraction were in fractions 8-10. Lipid rafts isolated from vehicle (PBS) treated mice were used to blot flotillin-1.

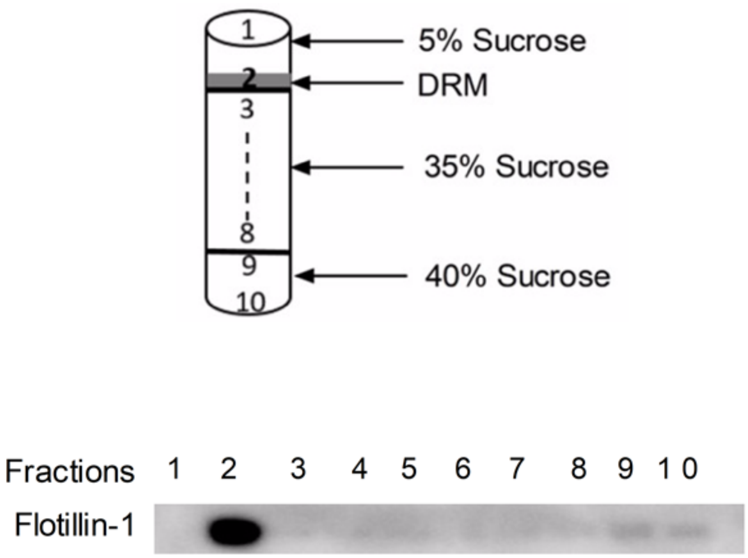

Fig. S3. Characterization of amyloidogenic proteins and other proteins in the ten fractions after lipid raft isolation. Fraction 2 was used for immunoblotting of amyloidogenic proteins and other proteins in lipid rafts.

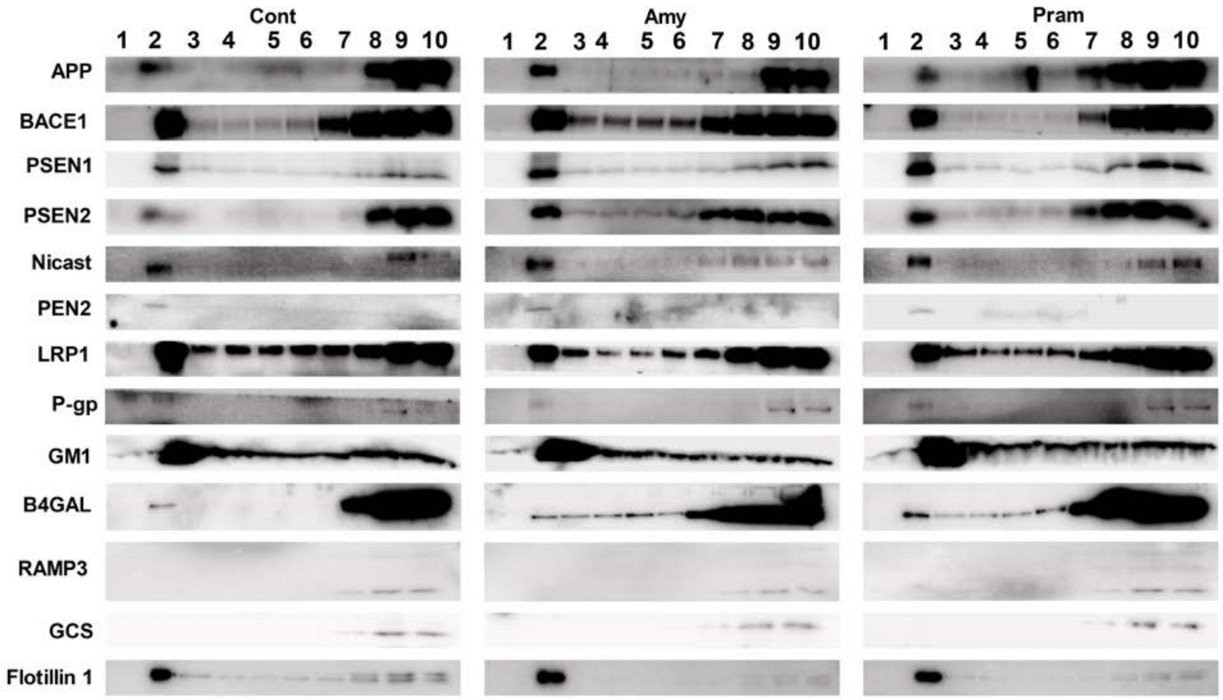

Fig. S4. Amylin and pramlintide did not alter expression of α-secretase as determined by Western blot in total brain homogenate **(A)**, while in lipid rafts in fraction 2, α-secretase and β-CTF were not detected using the separation conditions used in this study **(B).**

A

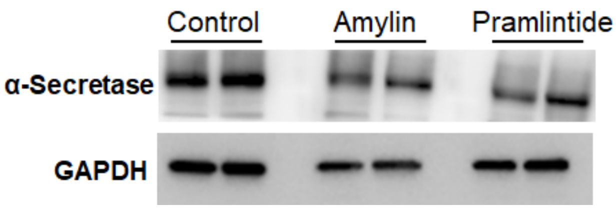

B

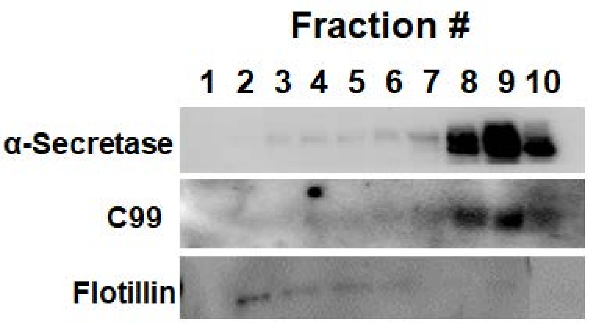

Fig. S5. Amylin and pramlintide did not alter expression of the three isoforms of RAMP3 (monomers, homodimers, and heterodimers) as determined by Western blot **(A)**, and quantification **(B)**. For RAMP3 detection, commericially avialable stain free kit (Bio-Rad) was used. Data is presented as mean±SEM for n=6/group.

A

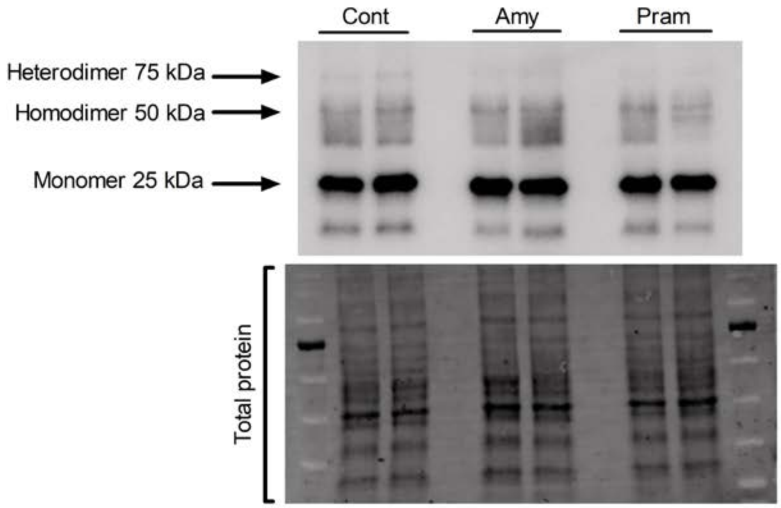

B

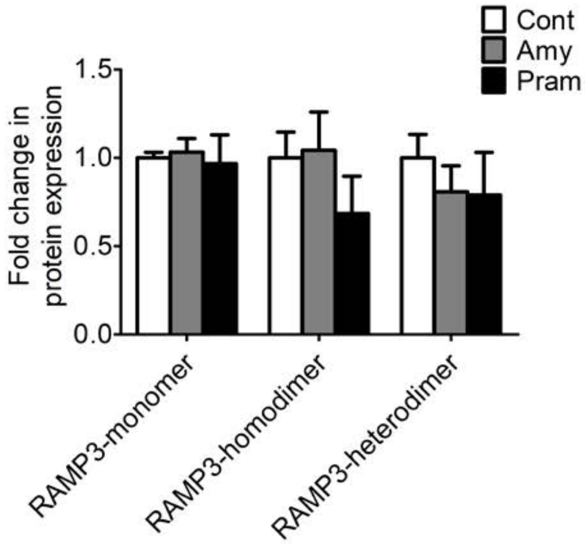

Fig. S6. Co-localization of hAPP and Cholera Toxin Subunit B (CTx-B) as shown by immunochemistry in wild-type C57Bl/6 **(A)**, and TgSwDI **(B-D)** mice brains. Brain sections were stained with a combination of Alexa Fluor 594 conjugated CTx-B (red; Thermo Fisher Scientific, 1:300 dilution) and anti-APP (A4; green; 1:300 dilution) as primary antibody for 2 h, and then with 1gG-Alexa Fluor 488 as secondary antibody (for 2 h), and analyzed by fluorescence microscopy. **(A)** Representative image from wild-type mouse brain stained with CTx-B showing GM1-enriched membrane lipid rafts (orange arrows); the section was stained with anti-APP, which was not detected in wild-type mouse brain. **(B)** Representative image from the brain of TgSwDI mouse received vehicle treatment stained with anti-APP (green) and CTx-B (red) showing an association of GM1with hAPP. **(C)** Representative image from the brain of TgSwDI mouse received amylin treatment stained with anti-APP (green) and CTx-B (red) showing an association of GM1with APP. **(D)** Representative image from the brain of TgSwDI mouse received pramlintide treatment stained with anti-APP (green) and CTx-B (red) showing increased association of GM1with APP compared to vehicle and amylin treatment groups. White arrows indicate co-localization. All images were captured at x40 fluorescence microscopy. Scale bar = 30 µm.

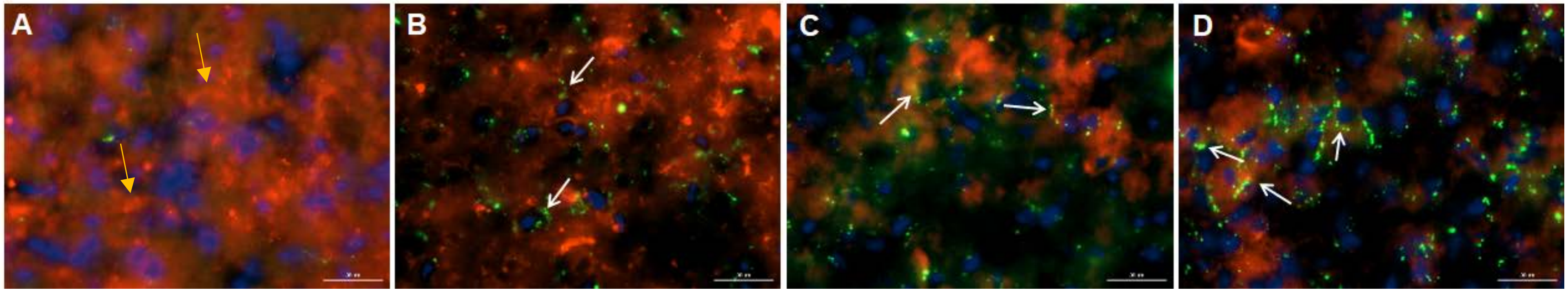

Supplement: Supplementary file 1 — Supplementary information [file 41598_2020_60664_MOESM1_ESM.pdf]
